# Psychopathological Concomitants and Motivations Related to Misuse of Non‐Steroidal Anti‐Inflammatory Drugs and Paracetamol

**DOI:** 10.1002/hup.70043

**Published:** 2026-04-23

**Authors:** Francesco Saverio Bersani, Elisabeth Prevete, Tommaso Accinni, Simone Montaldo, Irene Pinucci, Annalisa Maraone, Lorenzo Tarsitani, Massimo Pasquini, Claudio Imperatori

**Affiliations:** ^1^ Department of Medico‐Surgical Sciences and Biotechnologies Sapienza University of Rome Rome Italy; ^2^ Department of Human Neurosciences Sapienza University of Rome Rome Italy; ^3^ Department of Health and Life Sciences European University of Rome Rome Italy

**Keywords:** adverse childhood experiences, dysmorphic symptoms, mental health, non‐steroidal anti‐inflammatory drugs, paracetamol, psychological stress, substance misuse

## Abstract

**Introduction:**

Non‐steroidal anti‐inflammatory drugs (NSAIDs) and paracetamol are among the most commonly used medications worldwide. It has been suggested that the misuse of such medications is not uncommon. The objective of the present research was to explore prevalence, reported reasons and psychopathological concomitants related to NSAIDs/paracetamol misuse in a sample of users.

**Methods:**

The sample was made of 346 NSAIDs or paracetamol users (age range: 18–64 years); assessments included the 4‐item Perceived Stress Scale (PSS‐4), the 10‐item version of the Adverse Childhood Experiences International Questionnaire (ACE‐IQ‐10), the Body Image Concern Inventory (BICI), the Alcohol Use Disorders Identification Test‐C (AUDIT‐C), and questions related to NSAIDs/paracetamol misuse.

**Results:**

Forty‐eight individuals (13.9%) were categorized as NSAIDs/paracetamol misusers. A logistic regression model showed that higher scores in AUDIT‐C, BICI and ACE‐IQ‐10, and the use of psychiatric medications, were significantly associated with the likelihood of NSAIDs/paracetamol misuse. Specific reasons related to the misuse are also reported.

**Discussion:**

Our findings provide novel insights into the relationship between NSAIDs/paracetamol misuse and psychopathology, with potential clinical implications for prevention and treatment.

## 1 Introduction

Non‐steroidal anti‐inflammatory drugs (NSAIDs) and paracetamol are among the most commonly used medications worldwide, and their misuse is not uncommon (Algarni et al. 2021; Bloukh et al. 2021; Brennan et al. 2021). Circumstances related to misuse of NSAIDs or paracetamol include: polydrug use for recreational purposes (Bloukh et al. 2021), persistent use to treat pain (Algarni et al. 2021), management of tiredness, stressful situations or discomfort (Wojta‐Kempa and Krzyzanowski 2016), self‐inducing vomiting to control weight (Tiller and Treasure 1992), conditions related to addiction states (Brennan et al. 2021), and improvement of sport performance (Brennan et al. 2021). A potential involvement of NSAIDs in addiction‐related disturbances is also mentioned in the Fifth Edition of the Diagnostic and Statistical Manual of Mental Disorders (DSM5), within the section of “Other (or Unknown) Substance‐Related Disorders” (American Psychiatric Association 2013).

Overall, increasing data indicate medications misused in addition to, or in place of, other substances (Hernandez and Nelson 2010). Among the driving factors, medications have been reported to be misused as an attempt to improve performance or body image (McVeigh et al. 2012), as part of polydrug abuse behaviors (i.e., with medications being taken in addition to, or in place to, other substances in order to modulate the desired mental states) (McCabe et al. 2006), or as a strategy to cope with psychological distress (Bottiroli et al. 2018), including distress associated with forms of adverse childhood experiences (ACEs) (Beatty et al. 2024). Given these phenomena related to heterogeneous classes of medications, we aimed at testing whether similar patterns occur in relation to the misuse of NSAIDs or paracetamol.

Exploring the psychopathological underpinning of NSAIDs/paracetamol misuse is relevant in terms of prevention and treatment, also in light of the potential adverse effects of these medications. Considering the above, in the present study we aimed at preliminarily exploring (i) prevalence of NSAIDs/paracetamol misuse in a sample of adults from the general population, (ii) driving reasons associated to NSAIDs/paracetamol misuse, and (iii) psychopathological concomitants related to the areas of psychological stress (with focus on early traumas and perceived emotional discomfort), polydrug use (with focus on alcohol, cigarettes and illicit substances), and body dissatisfaction.

## 2 Methods

The current study included 346 adults (mean age: 36.11 ± 12.06 years; 72.8% females). Recruitment was performed through an online survey, disseminated with a non‐probability approach (snowball sampling) through web‐based tools (e.g., social media, instant messaging). Inclusion criteria for the present study were: (i) having declared to have used NSAIDs or paracetamol in the last 12 months, (ii) age between 18 and 64 years, (iii) good ability to understand written Italian, (iv) provision of correct response to two out of two questions of attentional quality check, and (v) provision of informed consent within the online survey. This study is part of a research project focused on medications and substances misuse in the general population approved by the Ethics Committee of European University of Rome (Prot. n. 02/2023).

A statistical power analysis was performed to determine the sample size using G*Power 3.1 software (Faul et al. 2009). The sample size requirement for the present study was chosen based on a non‐parametric between‐group analysis (i.e., our primary analysis) aimed at investigating differences between “misusers” and “non‐misusers”. Using a significance level of 0.05, the software estimated that a total sample size of 178 (i.e., 45 misusers and 133 non‐misusers) yield 80% power to detect a medium effect size (*d* = 0.50) in a two‐tailed test, assuming an allocation ratio of N2/N1 = 3. This allocation ratio was chosen based on studies reporting prevalence rates of inappropriate use of NSAIDs or over‐the‐counter analgesics of 19% (Cryer et al. 2016), 22% (Elander et al. 2014), 24% (Mehuys et al. 2012), 26.6% (Alharbi et al. 2020), and 32% (Kovac et al. 2010), thus we hypothesized that approximately 25% of NSAIDs/paracetamol users may engage in misuse.

For the present study, all participants were administered a checklist assessing certain clinical and socio‐demographic information (Table 1). Questions were asked investigating (i) whether NSAIDs/paracetamol were taken at larger dosages than suggested by the referring doctor or by the medication information sheet, (ii) whether NSAIDs/paracetamol were taken for a longer duration than suggested by the referring doctor or by the medication information sheet, and (iii) whether people in the participants' environment expressed complains for their use of the medication(s). If a person answered yes to one of these three questions (which reflect, to some extent, factors related to excessive use or functional impairment), he/she was considered as a NSAIDs/paracetamol misuser. Additional questions were asked to misusers exploring potential motives of misuse: achieving “high”/intense pleasure, improving performances in sport, improving physical appearance, or improving performances in study/work. A description of the specific questions is provided in Supporting Materials.

**TABLE 1:** Differences between groups, results of the logistic regression model.

	NSAIDs/paracetamol misusers (*n* = 48)	NSAIDs/paracetamol non misusers (*n* = 349)	Statistical test	*p*‐value	Beta	*p*‐value	OR	[95% CI] lower—upper
Age (years)—M ± SD	37.54 ± 11.62	35.88 ± 12.13	*U* = 6392.0	0.237	—	—	—	—
Female sex—*n* (%)	37 (77.1)	215 (72.1)	Chi^2^ = 0.290	0.590	—	—	—	—
BMI—M ± SD	23.26 ± 4.56	23.49 ± 4.74	*U* = 7068.5	0.896	—	—	—	—
N of workouts per week—M ± SD	1.17 ± 1.87	1.25 ± 1.66	*U* = 6735.0	0.467				
Having an academic degree—*n* (%)	16 (33.3)	146 (49.0)	Chi^2^ = 3.467	0.063	—	—	—	—
Students—*n* (%)	12 (25.0)	92 (30.9)	Chi^2^ = 0.428	0.513	—	—	—	—
Psychiatric medications use—*n* (%)	25 (52.1)	89 (29.9)	Chi^2^ = 0.8.259	**0.004**	**0.734**	**0.039**	**2.083**	**1.039–4.175**
Tobacco use—*n* (%)	24 (50.0)	97 (32.6)	Chi^2^ = 4.795	**0.029**	0.110	0.759	1.117	0.553–2.256
AUDIT‐C total score—M ± SD	3.81 ± 3.02	2.48 ± 2.13	*U* = 5301.5	**0.004**	**0.161**	**0.018**	**1.175**	**1.029–1.342**
Substances use (n)^a^—M ± SD	18 (37.5)	95 (31.9)	Chi^2^ = 0.366	0.545	—	—	—	—
PSS‐4 total score—M ± SD	8.44 ± 4.39	7.11 ± 3.77	*U* = 5892.5	**0.039**	−0.045	0.371	0.956	0.866–1.055
ACE‐IQ‐10 total score—M ± SD	3.29 ± 2.36	2.04 ± 1.93	*U* = 4921.0	**< 0.001**	**0.173**	**0.038**	**1.189**	**1.010–1.400**
BICI total score—M ± SD	58.23 ± 20.11	45.39 ± 16.63	*U* = 4471.0	**< 0.001**	**0.035**	**0.001**	**1.036**	**1.015–1.057**

*Note:* In bold significant values; Logistic regression model statistics: Chi^2^ = 40.236; DF = 6; *p* < 0.001; Nagelkerke *R* ^2^ = 0.20.

Abbreviations: ACE‐IQ‐10, 10‐item version of the Adverse Childhood Experiences International Questionnaire; AUDIT‐C, Alcohol Use Disorders Identification Test‐C; BICI, Body Image Concern Inventory; BMI, body mass index; CI, confidence interval; M, mean; NSAIDs, Non‐steroidal anti‐inflammatory drugs; OR, odds ratio; PSS‐4, 4‐items Perceived Stress Scale; SD, standard deviation.

^a^ Substace use refers to the reported recent use of cannabis, cocaine, heroin, cathinones or synthetic cannabinoids; individuals who answered “yes” or “maybe” in relation to their use of synthetic cannabinoids and cathinones were considered as users.

The Italian versions of 4‐item Perceived Stress Scale (PSS‐4) (Mondo et al. 2021), 10‐item version of the Adverse Childhood Experiences International Questionnaire (ACE‐IQ‐10) (Felitti et al. 1998), Body Image Concern Inventory (BICI) (Littleton et al. 2005; Luca et al. 2011), and Alcohol Use Disorders Identification Test‐C (AUDIT‐C) (Babor et al. 2001; Scafato et al. 2010) were also administered. A description of the scales is provided in Supporting Materials.

The Statistical Package for the Social Sciences version 29 (SPSS) was used for analyses. Data are expressed as means ± standard deviations (SD), or as percentages. For inferential analyses, statistical significance was set at *p* < 0.05. Considering the non‐normal distribution of several variables, the Mann‐Whitney test or chi squared test with Yates correction were used to evaluate between‐group differences for continuous and dichotomous variable, respectively.

As a supplementary analysis, all the variables significantly different between the groups of misusers and non misusers were inserted as independent variables in a logistic regression analysis with the misuser/non misuser condition as the dependent variable. The association is reported as odds ratio (OR), Beta coefficient and their *p*‐values.

## 3 Results

Based on the described questions, 48 individuals (13.9%) were categorized as NSAIDs/paracetamol misusers. Among them, four (8.3%) declared to have used such medication(s) to achieve “high”/intense pleasure, one (2.1%) to increase performances in sport competitions, two (4.2%) to improve physical appearance, three (6.3%) to improve performances in study/work. Between‐groups differences are reported in Table 1.

When comparing misusers (*n* = 48) and non misusers (*n* = 298) (Table 1), use of psychiatric medications, tobacco use, AUDIT‐C score, PSS‐4 score, ACE‐IQ‐10 score, and BICI score were significantly higher among misusers than among non misusers (all *p* ≤ 0.039). The logistic regression model was significant (Chi^2^ = 40.236, df = 6, *p* < 0.001) explaining 20% of the variability of binary dependent variable (i.e., NSAIDs/paracetamol misuser/non‐misuser condition). In this model, use of psychiatric medications, AUDIT‐C, BICI and ACE‐IQ‐10 total score were found to be significantly associated with the likelihood of NSAIDs/paracetamol misuse (Table 1).

## 4 Discussion

The present study gathered data on the psychopathological concomitants and motivations related to misuse (in terms of excessive use or dysfunctional use) of NSAIDs/paracetamol among individuals reporting the recent use of such medications.

Among the participants, 13.9% reported forms of misuse; this rate is lower than the rate observed in previous studies on the topic (Alharbi et al. 2020; Cryer et al. 2016; Elander et al. 2014; Kovac et al. 2010; Mehuys et al. 2012); such discrepancy may be due to various factors, including different conceptualizations of misuse, different medicines evaluated, and different characteristics of the samples. Overall, our data are consistent with previous evidence highlighting the potential dysfunctional use of NSAIDs and paracetamol (Algarni et al. 2021; Bloukh et al. 2021; Brennan et al. 2021) and contribute to raise awareness on this phenomenon. Mentioned motives for misuse included achievement of “high”/intense pleasure, improvements in sport, in physical appearance, and in work/study performances, supporting the possibility that people may take these medications to achieve desired effects which are different from the expected areas of clinical intervention.

Our findings also showed that certain psychopathological factors are significantly associated with the state of NSAIDs/paracetamol misuse: at multivariate level, in fact, increased adverse experiences during childhood, increased dysmorphia‐related feelings, use psychiatric medications, and increased alcohol use were associated with a higher likelihood of NSAIDs/paracetamol misuse.

It is possible that the relationship between the severity of body image concern and NSAIDs/paracetamol misuse may involve attempts to manage pain during excessive exercise or attempts to address perceived physical symptoms; this would be consistent with evidence suggesting that individuals with dysmorphic symptoms can inappropriately use substances (including medications) to improve perceived physical appearance (Cinaroglu and Yilmazer 2025; Hildebrandt and Alfano 2012; Nagata et al. 2022; Tiller and Treasure 1992), and with observations suggesting that certain fitness‐related social media trends (e.g., “fitspiration”) can in some cases favor dysfunctional use of medications or supplements (Cataldo et al. 2021).

In relation to the observed association between NSAID/paracetamol misuse and ACEs, it is well established that early traumatic experiences not only can affect mental health, but they can also have enduring effects on physical health, including manifestations at the sensorimotor level, such as patterns of pain, tension, and other bodily sensations (Felitti et al. 1998; Nicolson et al. 2023; van der Kolk 1994). ACEs are related to increased rates of misuse of certain medications during adulthood (Beatty et al. 2024; Merrick et al. 2020; M.T. Merrick et al. 2019). It is thus possible that our findings can be explained by these phenomena. The higher levels of ACEs in NSAIDs/paracetamol misusers could also partially explain the higher use of psychiatric medications in this group; indeed, it is known that ACEs are a significant risk factor for the development of mental disorders (Abate et al. 2025).

At the speculative level, it is also possible that NSAIDs/paracetamol medications are misused to cope with dysmorphia‐related or ACEs‐related emotional distress. Accordingly, a previous study observed that the non‐medical purposes for using over‐the‐counter pain relievers included “stressful situations” (Wojta‐Kempa and Krzyzanowski 2016); further, there is preliminary evidence that paracetamol and certain NSAIDs can in some cases contribute to alleviate psychopatological symptoms (Dewall et al. 2010; Fitton et al. 2022; Vangelisti et al. 2014). The issue, however, is complex and unclear, and it should interpreted also in light of evidence on potential negative effects of NSAIDs on psychiatric symptoms or on psychiatric interventions (Gallagher et al. 2012; Onder et al. 2004).

Lastly, the observed association of NSAIDs/paracetamol misuse with increased alcohol‐related problems supports the possibility that the misuse occur among individuals with dysfunctional use of other substances (including alcohol), consistently with the poly‐drug use behavior previously observed in relation to paracetamol as well as to various medications (Bloukh et al. 2021; McCabe et al. 2006). The use of illicit substances, however, was not associated with NSAIDs/paracetamol misuse in our study, and further research is warranted on the topic.

Several factors should be taken into consideration in the interpretation of the findings. Information on NSAIDs/paracetamol was collected through questions which were not previously validated. Self‐report measures intrinsically expose to potential biases (although we included only individuals who correctly answered to two attentional control questions). Furthermore, we did not evaluate distinctly the psychopathological concomitants of NSAIDs and paracetamol, nor the subtype of NSAIDs used. Fourth, this is a cross‐sectional report, thus it is cannot unequivocally establish the cause‐effect relationship between the study variables. The sample is also a convenience sample, not representative of sociodemographic features of Italian population. Lastly, not all the potential forms of drug misuse have been investigated.

In conclusion, this is to the best of our knowledge, the first study specifically testing through psychometric assessments the severity of psychopathological factors related to NSAIDs/paracetamol misuse. The findings provide novel pieces of insight on individuals and psychopathology associated with NSAIDs/paracetamol misuse, underlining potential implications for prevention and clinical management.

## Author Contributions

Conceptualization: F.S.B., C.I., interpretation of the data: F.S.B., C.I., E.P., T.A., S.M., I.P., A.M., L.T., M.P., statistical analysis: F.S.B., C.I., writing original draft: F.S.B., C.I., critical revisions of the manuscript: F.S.B., C.I., E.P., T.A., S.M., I.P., A.M., L.T., M.P., supervision: F.S.B., C.I., L.T., M.P.

## Funding

The authors have nothing to report.

## Ethics Statement

This study is part of a research project focused on medications and substances misuse in the general population approved by the Ethics committee of European University of Rome (Prot. n. 02/2023).

## Consent

One of the inclusion criteria for the present study was provision of informed consent within the online survey.

## Conflicts of Interest

The authors declare no conflicts of interest.

## Supporting information


Supporting Information S1

## Data Availability

Aggregated data may be available from the corresponding authors upon reasonable requests.
