# Supplementary material for: Psychopathological Concomitants and Motivations Related to Misuse of Non‐Steroidal Anti‐Inflammatory Drugs and Paracetamol
Source: Hum Psychopharmacol. 2026 Apr 23;41:e70043. doi: 10.1002/hup.70043 (PMC13104796; doi:10.1002/hup.70043)
Supplement: Supplementary file 1 — Supporting Information S1 [file HUP-41-e70043-s001.docx]

**SUPPLEMENTARY MATERIALS**

**Question on NSAIDs/paracetamol use (allowed answers: yes/no)**

• *Original question*: Negli ultimi 12 mesi Le è capitato di assumere un farmaco (o più farmaci) anti-dolorifico e/o anti-infiammatorio non steroideo (ad esempio paracetamolo, ibuprofene, naprossene, acido acetilsalicilico, nimesulide, ketoprofene)?

Risponda “Sì” se Lei, negli ultimi 12 mesi, ha utilizzato tale/i medicinale/i per qualunque motivazione (motivazioni di tipo clinico/sanitario, di tipo ricreativo, o di altra natura), in qualunque modalità (nelle modalità previste dalla scheda tecnica o in modalità diverse), in qualunque dosaggio, per qualsiasi durata (in una singola circostanza, occasionalmente, o in maniera protratta nel tempo). Risponda “No” se Lei, negli ultimi 12 mesi, non ha mai, in nessuna circostanza,utilizzato tale/i medicinale/i.

*English translation*: In the last 12 months, did you take one (or more) analgesic and/or anti-inflammatory nonsteroidal drug (for example: paracetamol, ibuprofen, naproxen, acetylsalicylic acid, nimesulide, ketoprofen)?

Answer “Yes” if, in the last 12 months, you did take this/these medication/medications for any reason (for health reasons, for recreational reasons, or for other reasons), with any modality (in the modality described in the medication information sheet or in different modality), at any dosage, with any duration (in one single circumstance, occasionally, or repeatedly over time). Answer “No” if, in the last 12 months, you have never, in no case, used this/these medication/medications

**Questions on NSAIDs/paracetamol misuse (allowed answers: yes/no)**

• *Original question*: Negli ultimi 12 mesi Le è capitato di assumere questo/i farmaco/i della famiglia degli anti-dolorifici e/o anti-infiammatori non steroidei in quantità maggiori rispetto a quanto suggerito dal medico di riferimento o rispetto a quanto indicato nella scheda tecnica?

*English translation*: In the last 12 months did you take this/these analgesic and/or nonsteroidal anti-inflammatory drug/drugs at larger dosages than suggested by the referring doctor or by the medication information sheet?

• *Original question*: Negli ultimi 12 mesi Le è capitato di assumere questo/i farmaco/i della famiglia degli anti-dolorifici e/o anti-infiammatori non steroidei per più tempo rispetto a quanto suggerito dal medico di riferimento o rispetto a quanto indicato nella scheda tecnica?

*English translation*: In the last 12 months did you take this/these analgesic and/or nonsteroidal anti-inflammatory drug/drugs for a longer duration than suggested by the referring doctor or by the medication information sheet?

• *Original question*: Le è capitato, negli ultimi 12 mesi, che qualcuno nel suo ambito familiare, amicale o lavorativo si sia lamentato per il suo uso di questo/i farmaco/i della famiglia degli anti-dolorifici e/o anti-infiammatori non steroidei?

*English translation*: In the last 12 months, did someone in your family, among your friends, or in your work environment, express complains for your use of this/these analgesic and/or nonsteroidal anti-inflammatory drug/drugs?

**Questions exploring potential driving motives related to NSAIDs/paracetamol misuse (allowed answers: yes/no)**

• *Original question*: Le è capitato, negli ultimi 12 mesi, di assumere questo/i farmaco/i della famiglia degli anti-dolorifici e/o anti-infiammatori non steroidei per ottenere unasensazione di “sballo” o di intenso piacere?

*English translation*: In the last 12 months did you take this/these analgesic and/or nonsteroidal anti-inflammatory drug/drugs to achieve feelings of “high” or of intense pleasure?

• *Original question*: Le è capitato, negli ultimi 12 mesi, di assumere questo/i farmaco/i della famiglia degli anti-dolorifici e/o anti-infiammatori non steroidei per migliorare le performance in una competizione sportiva agonistica?

*English translation*: In the last 12 months did you take this/these analgesic and/or nonsteroidal anti-inflammatory drug/drugs to increase performances in agonistic sport competitions?

• *Original question*: Le è capitato, negli ultimi 12 mesi, di assumere questo/i farmaco/i della famiglia degli anti-dolorifici e/o anti-infiammatori non steroidei per fini estetici e/o per migliorare l’aspetto fisico?

*English translation*: In the last 12 months did you take this/these analgesic and/or nonsteroidal anti-inflammatory drug/drugs for aesthetic reasons and/or to improve physical appearance?

• *Original question*: Le è capitato, negli ultimi 12 mesi, di assumere questo/i farmaco/i della famiglia degli anti-dolorifici e/o anti-infiammatori non steroidei per eccellere e/o primeggiare nello studio o nel lavoro?

*English translation*: In the last 12 months did you take this/these analgesic and/or nonsteroidal anti-inflammatory drug/drugs to excel in study or work?

**Measures**

The PSS-4 is a short version of the PSS questionnaire; it consists of 4 items scored on a 0-4 scale, with a total score scores ranging from 0 to 16, and with a higher score indicating higher levels of perceived stress [1]; Chronbach’s alpha in the present sample was 0.797.

The ACE-IQ-10 is made of 10 groups of questions with possible “yes” or “no” answers, investigating the occurrence during childhood of various forms of adverse events; scores range between 0 and 10, with a higher score indicating higher presence of adverse events [2]; Chronbach’s alpha in the present sample was 0.706.

The BICI is a self-report tool assessing dysmorphia-related symptoms, consisting of 19 items scored on a 1-5 scale; items are related to various aspects of dysmorphia; scores can range from 19 to 95; higher scores suggest more severe dysmorphic concerns [3-4]; Chronbach’s alpha in the present sample was 0.945.

The AUDIT-C questionnaire is a short version of the AUDIT questionnaire, which was developed by the World Health Organization (WHO) as an assessment tool for excessive drinking. It includes three questions; items are scored on a 0-4 scale; higher scores suggest more severe alcohol misuse [5-6]. Chronbach’s alpha in the present sample was 0.734.

**References**

1. Mondo, M., C. Sechi, and C. Cabras, *Psychometric evaluation of three versions of the Italian Perceived Stress Scale.* Curr Psychol 2021. **40**: p. 1884–1892.

2. Felitti, V.J., et al., *Relationship of childhood abuse and household dysfunction to many of the leading causes of death in adults. The Adverse Childhood Experiences (ACE) Study.* Am J Prev Med, 1998. **14**(4): p. 245-58.

3. Littleton, H.L., D. Axsom, and C.L. Pury, *Development of the body image concern inventory.* Behav Res Ther, 2005. **43**(2): p. 229-41.

4. Luca, M., et al., *Measuring dysmorphic concern in Italy: psychometric properties of the Italian Body Image Concern Inventory (I-BICI).* Body Image, 2011. **8**(3): p. 301-5.

5. Scafato, E., et al., *L’alcol e l’assistenza sanitaria primaria. Linee guida cliniche per l’identificazione e l’intervento breve*. 2010.

6. Babor, T.F., et al., *The Alcohol Use Disorders Identification Test Guidelines for Use in Primary Care*. 2001: World Health Organization.
